# Effects of QiShenYiQi dropping pills on gut dysbiosis and statin-associated muscle symptoms in ApoE^−/−^ mice with type 2 diabetes mellitus and coronary heart disease

**DOI:** 10.3389/fcell.2026.1837554

**Published:** 2026-07-27

**Authors:** Jingxin Zhang, Bingzhen Shang, Zheng Wang, Chenyu Jiang, Peng Zhang, Tiancheng Xu, Jing-Yan Han, Yin Li

**Affiliations:** 1 Department of Integration of Chinese and Western Medicine, School of Basic Medical Sciences, Peking University, Beijing, China; 2 Tasly Microcirculation Research Center, Peking University Health Science Center, Beijing, China; 3 The Key Discipline for Integration of Chinese and Western Basic Medicine (Microcirculation) of the National Administration of Traditional Chinese Medicine, Beijing, China; 4 Key Laboratory of Stasis and Phlegm, State Administration of Traditional Chinese Medicine of the People’s Republic of China, Beijing, China; 5 Beijing Microvascular Institute of Integration of Chinese and Western Medicine, Beijing, China; 6 Department of Clinical Trial Ward, Clinical Trial and Conversion Center, Shengjing Hospital of China Medical University, Shenyang, Liaoning, China; 7 Peking University Third Hospital, Peking University Health Science Center, Beijing, China; 8 Key Laboratory of Acupuncture and Medicine Research of Ministry of Education, Nanjing University of Chinese Medicine, Nanjing, Jiangsu, China

**Keywords:** coronary heart disease, gut metabolites, gut microbiota, qishenyiqi dropping pills, statin-associated muscle symptoms, transcripomics, type 2 diabetes mellitus

## Abstract

**Background:**

Statins are first-line therapeutic agents for metabolic syndrome, but their muscular adverse effects increase the metabolic burden in Type 2 diabetes mellitus (T2DM) and coronary heart disease (CHD). QiShenYiQi dropping pills (QSYQ), a compound Chinese medicine, exerts cardioprotective and metabolic regulatory effects. However, whether it is beneficial to gut microbiota dysbiosis and statin-associated muscle symptoms (SAMS) remains unclear.

**Methods:**

The disease model with T2DM complicated with CHD was established in APOE^−/−^ mice. The mice were randomly divided into normal control (NC), disease model (DM), Simvastatin + Fenofibrate (SF), Simvastatin + Fenofibrate + QSYQ (SFQ), and Simvastatin + Fenofibrate + Trimetazidine (SFT) groups, with 90 days of intervention. Using 16S rRNA sequencing, untargeted metabolomics, and transcriptomics, we investigated the regulatory effects and mechanism of QSYQ on unbalanced microbiota composition and SAMS.

**Results:**

Combination therapy with QSYQ markedly ameliorated glycemic and lipid profiles in animals with comorbid T2DM and CHD, and outperformed other treatment strategies in preserving hepatic and skeletal muscle function. These beneficial effects may be correlated to the regulation of gut microbiota, glycolipid metabolites and associated gene expression, including inflammation-related genes, apoptosis-related genes, as well as antioxidant and energy metabolism-related genes.

**Conclusion:**

These findings suggested the potential value of QSYQ as a promising candidate for T2DM and CHD, attenuating the side effects of statins, providing a novel complementary strategy for clinical treatment.

## Graphical Abstract

**Figure:**
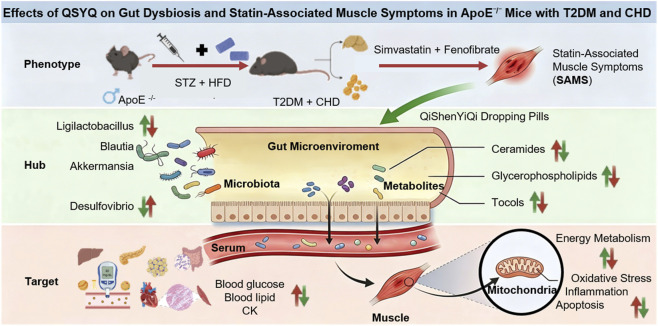

## 1 Introduction

Type 2 diabetes mellitus (T2DM) and coronary heart disease (CHD), as core comorbidities in the spectrum of metabolism-related diseases (Garcia-Fernandez et al., 2025), have long maintained high morbidity and mortality rates, emerging as a major global public health challenge and imposing a heavy burden on patients’ health and healthcare systems (Tian et al., 2025; Guzman-C et al., 2023). Epidemiological evidence confirms a tight pathological interaction between T2DM and CHD (Kuo and Li, 2025; Vujkovic et al., 2020), which mutually exacerbate insulin resistance, pancreatic dysfunction (Sandforth et al., 2025) and cardiovascular risk (Ferrannini et al., 2024). They share core pathogenic mechanisms: obesity-induced insulin resistance triggers inflammation, oxidative stress, endothelial injury and ectopic lipid deposition to drive atherosclerosis and β-cell dysfunction (Schlesinger et al., 2022). Hyperglycemia-derived advanced glycation end products and dyslipidemia further destabilize coronary plaques and accelerate disease progression (Bellemare et al., 2025). Early lipid-lowering treatment ameliorates endothelial function and plaque stability, thus alleviating glucose metabolic disorders (Lu et al., 2024).

Statins are first-line lipid-lowering agents (Wright and Subramanian, 2024), whereas fibrates exert both lipid-regulating and mild hypoglycemic effects (Holm et al., 2019). However, their combined use aggravates statin-induced hepatic, digestive and muscular damage (Freeman et al., 2006; Squizzato et al., 2010). Clinically, statin-associated muscle symptoms (SAMS), including myalgia, myositis and even rhabdomyolysis, have become increasingly prevalent (Vinci et al., 2021), highlighting the need for safer and more precise interventions.

QiShenYiQi dropping pills (QSYQ), a compound traditional Chinese medicine, is a standardized Chinese medicinal preparation approved by the China Food and Drug Administration (CFDA, approval number Z20030139), manufactured by Tasly Pharmaceutical Group Co., Ltd (Tianjin, China). It consists of four herbal materials (Astragali Radix (Huangqi), Salviae Miltiorrhizae Radix et Rhizoma (Danshen), Notoginseng Radix et Rhizoma (Sanqi), and Dalbergiae Odoriferae Lignum (Jiangxiang)). It exerts multiple pharmacological effects, including restoring energy metabolism and myocardial structure, improving cardiomyocyte apoptosis and mitochondrial function, and inhibiting inflammation (Li J. et al., 2025).

Single-disease animal models merely recapitulate either isolated diabetes or atherosclerosis, failing to replicate the authentic clinical profile of patients. To fill this gap, we constructed a compound T2DM-CHD model in ApoE^−/−^ mice, followed by co-administration of simvastatin and fenofibrate. This modeling system faithfully mirrors clinical dual lipid-lowering regimens, and simultaneously recapitulates both intrinsic pathological injuries stemming from metabolic comorbidity and superimposed myotoxicity triggered by combined lipid-lowering agents, thereby possessing promising translational potential for clinical research. Using this model, we further explored the protective effects of QSYQ via integrated analyses of gut microbiota, intestinal metabolome and skeletal muscle transcriptome, so as to provide a novel strategy for developing metabolism-targeted combined therapy.

## 2 Methods

### 2.1 Animals experiments

The 30 male ApoE^−/−^ mice (22 ± 2 g) were obtained from the Animal Center of Peking University Health Science Center. Mice were kept at a temperature of 23 °C ± 2 °C and relative humidity of 40% ± 5%, with a 12 h light/dark cycle. Animal experiments were conducted according to the guidelines of the Experimental Animal Center and were approved by the Medicine Experimental Animal Research Ethics Committee of the Peking University Health Science Center (Approval No. PUIRB-LA2022590).

**FIGURE 1:**
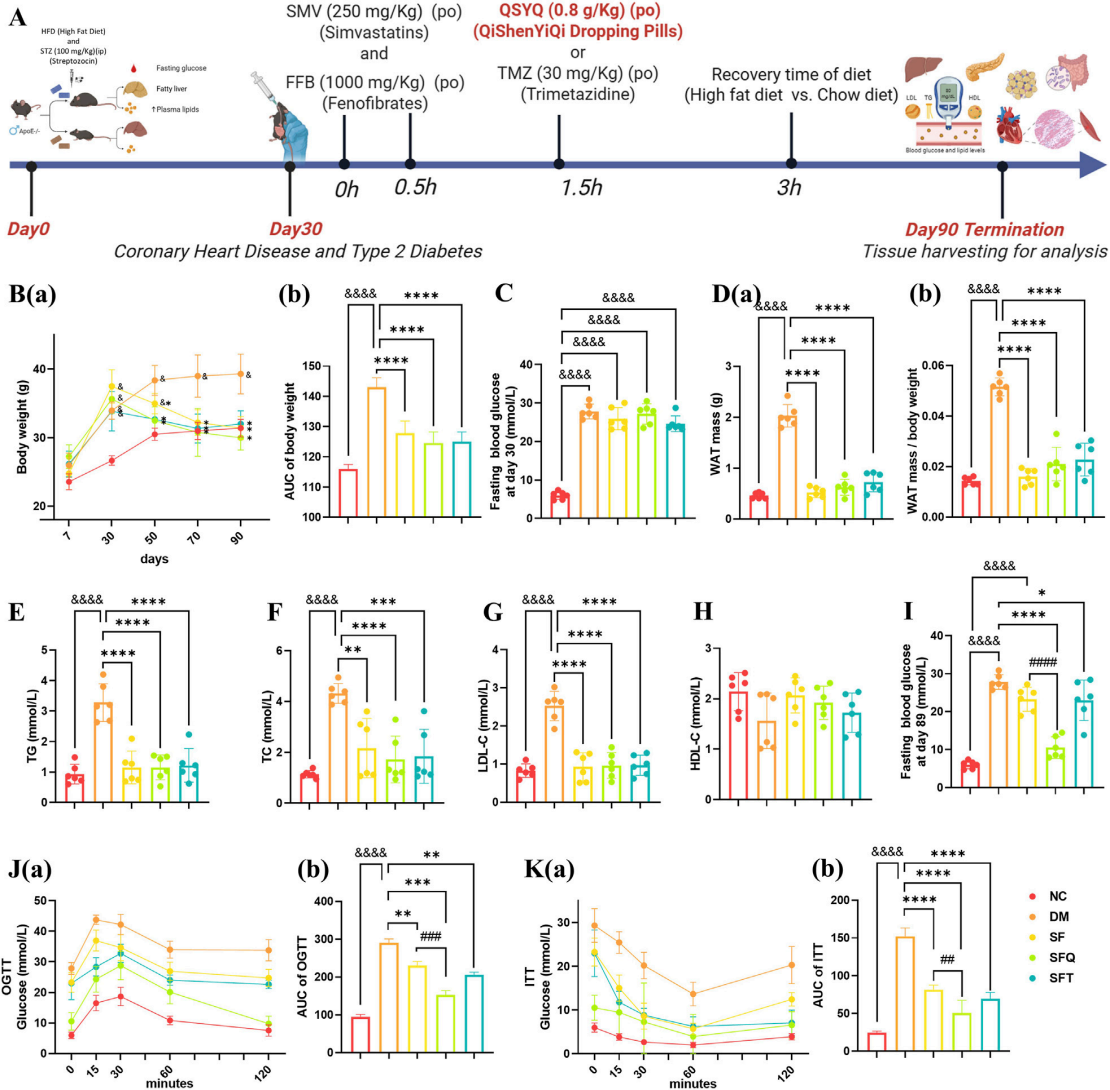
Statins and QSYQ improve metabolism related physiological parameters. **(A)** Schematic diagram showing the procedure of long-term administration of drugs for ApoE^−/−^ mice. **(B)** Body weight and area under the curve (AUC). **(C)** Measurements of fasting blood glucose at day 30 .**(D)** WAT mass and AUC. **(E–H)** Serum TG, TC, LDL-C, HDL-C. **(I)** Measurements of fasting blood glucose at day 89. **(J)** OGTT and AUC for ApoE^−/−^ mice at day 84. **(K)** ITT and AUC for ApoE^−/−^ mice at day 90.Six mice per group, all data are expressed as mean ± standard deviation. One-way analysis of variance (ANOVA) was used for statistical analysis. &*p* < 0.05, &&*p* < 0.01 compared with NC group, ^✱^ *p* < 0.05, ^✱✱^ *p* < 0.01 compared with DM group, #*p* < 0.05, ##*p* < 0.01 compared with SF group.

The mice were devided into five different groups (normal control (NC), disease model (DM), Simvastatin + Fenofibrate (SF), Simvastatin + Fenofibrate + QSYQ (SFQ) and Simvastatin + Fenofibrate + Trimetazidine (SFT)) and the specific treatments and dosages referred to Figure 1A and Supplementary Material (Heydemann, 2016; Gao et al., 2025).

### 2.2 Biochemical index analysis

Enzyme-linked immunosorbent assay (ELISA) biochemical markers kits (Andygene, Beijing, China) were used to detect the serum and tissue index (CK, AST, ALT, TG, TC, LDL-C, HDL-C) in mice, according to the instructions by using a microplate reader (Thermo Fisher Scientific Inc., Illinois, USA).

To perform the oral glucose tolerance test (OGTT) at day 84, the ApoE^−/−^ mice received a 2 g/kg glucose solution following a 12 h fasting period with *ad libitum* water access. And at day 90, an insulin tolerance test (ITT) was conducted with 1 U/ kg of insulin (Beyotime, Shanghai, China) i. p. Injected into the mice had fasted for 12 h. Both of the blood glucose levels were examined 0, 15, 30, 60 and 120 min after the administration. The curve of the blood glucose change was drawn, and the area under the curve (AUC) of OGTT or ITT was calculated using the corresponding formula.

### 2.3 Exercise endurance and maximum oxygen consumption test

A treadmill system (LE8700TS, Panlab/Harvard Apparatus, Barcelona, Spain) equipped with an indirect calorimetry module was used to evaluate exercise capacity and maximal oxygen consumption (VO_2_max). Prior to formal testing, all mice were subjected to a 3-day acclimatization period on the treadmill at a speed of 10–15 m/min for 10 min daily to minimize stress responses. The formal test adopted an incremental loading protocol: starting at 10 m/min, the speed was increased by 3 m/min every 3 min until exhaustion, defined as the inability to maintain the treadmill speed for more than 10 s despite mild electrical stimulation. The exhaustive time, exhaustive distance, maximum running speed, and VO_2_max were automatically recorded by the system software for subsequent analysis.

### 2.4 Grip strength measurement

A grip strength meter (YR02-YLS-13A, Yuyan Instruments Co., Ltd., Shanghai, China) was employed to assess skeletal muscle function in mice. Before testing, the device was calibrated and zeroed according to the manufacturer’s instructions. Each mouse was allowed to grasp the metal grid with its forelimbs, and then gently pulled backward in a horizontal plane until it released the grid; the peak force value was recorded automatically. Each mouse was tested five times with a 30-s interval between trials, and the average of three valid measurements was taken as the final grip strength (g) to reduce variability. All tests were performed at the same time of day to avoid circadian rhythm interference.

### 2.5 Histopathology

The skeletal muscle tissue were fixed in 4% paraformaldehyde solution, followed by dehydration, embedding, and sectioning. After baking and dewaxing, the muscle tissue sections were hydrated using a graded ethanol series. For H&E staining, the sections were stained with hematoxylin for 5 min followed by eosin for 5–10 s. For Periodic Acid-Schiff (PAS) staining to observe glycogen distribution, sections were oxidized in 0.5% periodic acid solution for 10 min, rinsed, and immersed in Schiff’s reagent in the dark for 15–20 min. After washing, sections were counterstained with hematoxylin. For dystrophin immunofluorescence staining, after antigen retrieval, blocking, and incubation with primary antibody against dystrophin, the sections were incubated with fluorescence-conjugated secondary antibody and nuclei were counterstained with DAPI. Images were captured and analyzed by BioTek Cytation7(Agilent Technologies, CA, USA).

### 2.6 16S rRNA sequencing and gut microbiota

16S rRNA Sequencing experiments were performed by Novogene (Beijing, China). Microbial DNA was extracted from mouse fecal samples, and the V4 region of 16S rRNA was amplified and sequenced on the NovaSeq 6000 platform. Raw sequencing data were processed for sequence assembly, quality control, and chimera removal, and further analyzed using QIIME2 to generate ASVs with taxonomic annotation. Bacterial diversity was evaluated by alpha and beta diversity analysis. Intergroup differences in microbiota composition were assessed using multiple statistical methods and functional prediction was performed using PICRUSt2.

### 2.7 Untargeted Metabolomics Sequencing

Untargeted Metabolomics Sequencing experiments were performed by Novogene (Beijing, China). The intestinal contents of mice in each group were collected for LC-MS analysis after grinding in liquid nitrogen, extraction with 80% methanol, and low-temperature centrifugation. An equal volume of each sample was mixed to prepare quality control (QC) samples. Based on the format conversion, peak extraction, correction and filtering of raw data, Novogene database was used to identify the metabolites. Principal component analysis (PCA), k-means clustering and correlation analysis were used to analyze the relationships among samples and metabolites. Furthermore, metabolic pathway analysis was performed to deduce the biological significance of the metabolites.

### 2.8 RNA-seq

RNA-seq experiments were conducted by Novogene (Beijing, China). Total RNA was extracted from muscle tissues of each group (5 biological replicates per group) using TRIzol reagent. After mRNA purification, sequencing libraries were constructed and subjected to 150 bp paired-end sequencing on the Illumina HiSeq platform. Raw data were quality-controlled and filtered to obtain clean reads, which were then aligned to the reference genome. The resulting P values were adjusted using the Benjamini–Hochberg method for controlling the false discovery rate. Genes with an adjusted P value <0.05 identified by DESeq2 were defined as differentially expressed genes.

### 2.9 Multi-omics correlation analysis

The sequencing data was first analyzed to evaluate the expression of key microbiota, metabolites and genes. Pearson correlation analysis was performed to calculate the correlation coefficients (r) and P values based on the relative abundances of all the differential gut microbia at the genus level and the quantitative values of all the differentially expressed metabolites and genes, using P < 0.05 and |r| > 0.5 as threshold parameters. Network diagrams were generated using the mixOmics package in R language.

### 2.10 qPCR

Total RNA from the mouse gastrocnemius muscle was isolated using TRIzol reagent (Invitrogen, Thermo Fisher Scientific, USA) according to the manufacturer’s instructions, and reverse transcription reaction was carried out with the specific enzyme (Yeasen Biotech, Shanghai, China). The mRNA expressions of Ccr2, Ppm1e, Hspa1a, Hspa1b, Fam107a, Zfp612, Fgfr3, Map3k6, Kcnd3 and Dbp were quantified by QuantStudio five real-time PCR system (Applied Biosystems, Thermo Fisher Scientific, USA) and each sample was prepared in a 20 µL reaction mixture containing 40 ng of cDNA, 10 µL of Universal SYBR Green Supermix (Yeasen Biotech, Shanghai, China), and 250 nM of both forward and reverse primers. The primer sequences used in this study were shown in Supplementary Table S1. The GAPDH primers were used as housekeeping control.

### 2.11 Statistical analysis

Bioinformatics analysis was performed using the R software. GraphPad Prism 9.0 software was used to analyze all experimental measurement data. The data were expressed as mean ± standard deviation (SD). The t-test was used for pairwise comparison when the data conformed to a normal distribution. For data with a non-normal distribution, Wilcoxon rank-sum test was used for pairwise comparison, and Bonferroni method was used for inter-group correction. P value <0.05 was considered as statistically significant and p value <0.01 was considered as highly statistically significant.

## 3 Results

### 3.1 QSYQ ameliorates the glucose and lipid metabolic phenotype in an ApoE^−/−^ mice model of comorbid T2DM and CHD

We established a comorbid T2DM and CHD model in ApoE^−/−^ mice using a classic combination of HFD and STZ, and administered simvastatin (SMV), a first-line clinical statin, in combination with fenofibrate (FFB) for the treatment of metabolic syndrome. However, long-term statin use imposes a heavy burden on the liver and kidneys and causes skeletal muscle adverse reactions. Therefore, we administered QSYQ, a compound traditional Chinese medicine, to ameliorate the side effects of statins, with trimetazidine (TMZ) as a positive control drug (Figure 1A). Our findings demonstrated that after successful model establishment (Figures 1B,C), the body weight (BW) of mice in the SF, SFQ, and SFT groups gradually decreased over time compared with the DM group (Figure 1B). Additionally, QSYQ and TMZ did not affect the lipid-lowering efficacy of statins, with significant reductions in white adipose tissue (WAT) mass and the WAT/BW ratio observed in all drug-administered groups (Figure 1D), as well as significant decreases in plasma total cholesterol (TC), triglycerides (TG), low-density lipoprotein cholesterol (LDL-C), and high-density lipoprotein cholesterol (HDL-C) (Figures 1E–H). Unlike the positive drug TMZ, QSYQ exhibited a favorable hypoglycemic effect. The fasting blood glucose (FBG) level (Figure 1I) in the SFQ group was significantly reduced, and the improvements in oral glucose tolerance test (OGTT) (Figure 1J) and insulin tolerance test (ITT) (Figure 1K) were significantly superior to those in the SF and SFT groups.

### 3.2 QSYQ attenuates statins associated muscle symptoms and liver dysfunction

**FIGURE 2:**
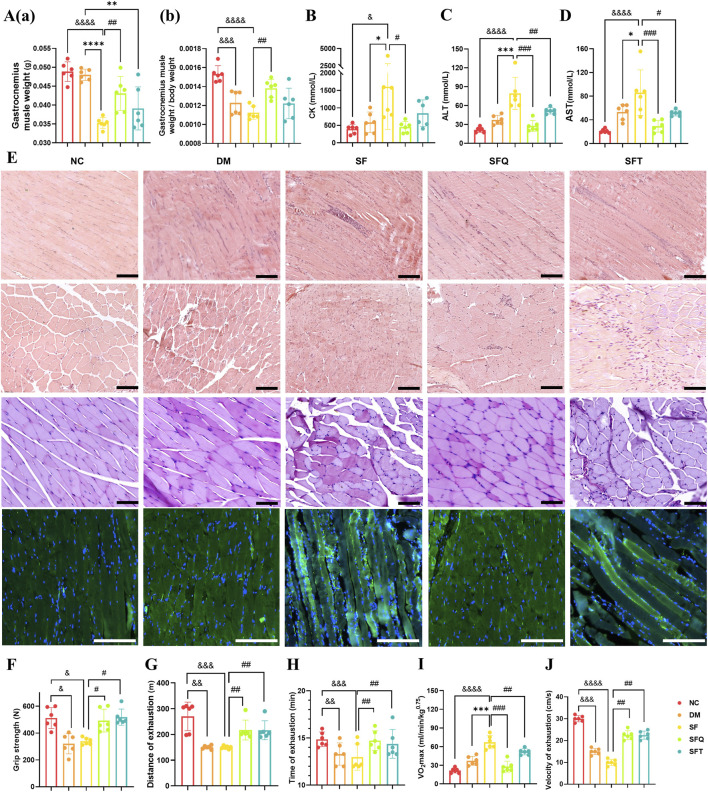
QSYQ improves biochemical indicators and skeletal muscle injury induced by statins. **(A)** Gastrocnemius muscle mass and AUC. **(B)** Serum creatine kinase (CK). **(C,D)** Serum ALT and AST **(E)** Representative images of H&E staining (Scale bars = 100 μm, transverse and longitudinal sections, line 1–2), PAS staining (Scale bars = 100 μm, line 3) and immunofluorescence of muscle injury-related proteins (Scale bars = 25 μm, Dystrophin, line 4). **(F)** Grip strength. **(G–J)** Exercise exhaustion tolerance. Six mice per group, all data are expressed as mean ± standard deviation. One-way analysis of variance (ANOVA) was used for statistical analysis. &*p* < 0.05, &&*p* < 0.01 compared with NC group, ^✱^ *p* < 0.05, ^✱✱^ *p* < 0.01 compared with DM group, #*p* < 0.05, ##*p* < 0.01 compared with SF group.

Our research systematically summarized evidence of statin-associated muscle injury from three dimensions: serum biochemical markers, skeletal muscle histopathology, and *in vivo* muscle functional assays. QSYQ significantly ameliorated statin-associated muscle symptoms (SAMS), effectively reversing the abnormal decrease in gastrocnemius mass and the gastrocnemius mass/BW ratio (Figure 2A), and the abnormal elevation of plasma creatine kinase (CK) levels (Figure 2B) in the SF group, while these effects were not observed with TMZ. Meanwhile, the liver function indices alanine aminotransferase (ALT) and aspartate aminotransferase (AST) (Figures 2C,D) were significantly improved in the SFQ and SFT groups compared with the SF group. Morphological studies further verified the beneficial effects of QSYQ on skeletal muscle (Figure 2E). Hematoxylin-eosin (HE) staining revealed varying degrees of inflammatory infiltration, as well as nuclear migration and fusion in skeletal muscle tissues of the DM and SF groups, and QSYQ exerted a significantly better ameliorative effect on these abnormalities than TMZ. Periodic acid-Schiff (PAS) staining showed abnormal glycogen deposition in skeletal muscle tissues of the DM and SF groups, and QSYQ had a less pronounced ameliorative effect on this than TMZ. Immunofluorescence analysis indicated that the muscle injury-related protein dystrophin was abnormally expressed in the SF group, and QSYQ significantly reversed this abnormality, while TMZ had no obvious effect.

Functional assessments of muscle performance and exercise capacity further supported these findings. Grip strength (Figure 2F), reflecting skeletal muscle function, was significantly reduced in the DM and SF groups, while both SFQ and SFT treatment improved grip strength, with QSYQ showing a more robust effect. Consistent with these observations, the SF group exhibited impaired exercise tolerance, as evidenced by reduced exhaustive distance (Figure 2G), shorter exhaustive time (Figure 2H), decreased maximal oxygen consumption (VO_2_-max, Figure 2I), and lower maximal exhaustive velocity (Figure 2J). Notably, QSYQ treatment significantly improved all these exercise-related parameters.

### 3.3 Analysis of α-diversity and β-diversity of intestinal flora in 5 groups of mice

We evaluated the alterations in intestinal flora structure and the regulatory effects of various therapeutic regimens in mice with comorbid T2DM and CHD through 16S rRNA gene V4 region sequencing of fecal samples. The α-diversity and β-diversity analyses were used to reflect the overall richness and diversity of the microbial community and to clarify the impact of comorbid T2DM and CHD on the overall structure of intestinal flora. The species accumulation box plot tended to plateau, indicating that the samples met the requirements for further analysis (Supplementary Figure S1A). The Rank–Abundance curve showed that the species richness of the SFQ group was higher than that of the SF group with a more uniform species distribution, while the DM group exhibited the poorest species richness (Supplementary Figure S1B). Compared with the NC group, the α-diversity indices of the DM and SF groups were significantly reduced, with the SF group showing the lowest chao1 index, suggesting impaired diversity and evenness of flora composition in this group; the SFQ group significantly modulated these abnormalities (Supplementary Figure S1C; Supplementary Figure S2A). β-Diversity intergroup difference tests revealed significant intergroup differences in the intestinal flora community composition of mice in each group, with high intra-group sample similarity (Supplementary Figure S1D). This characteristic was also suggested by the Bray–Curtis distance matrix heatmap based on dissimilarity coefficients (Supplementary Figure S2A). Principal coordinate analysis (PCoA), principal component analysis (PCA), and non-metric multidimensional scaling (NMDS) all showed intergroup separation of samples in each group from different dimensions (Supplementary Figure S1E; Supplementary Figure S2B,C).

Beta nearest taxon index (βNTI), normalized stochasticity ratio (NST), and the infer community assembly mechanisms by phylogenetic-bin-based null model analysis (iCAMP) were performed to elucidate the ecological processes governing microbial community structure and assembly, including selection, dispersal, and stochasticity (Supplementary Figure S2F-H).

### 3.4 Differences in intestinal flora community structure of 5 groups of mice

The unweighted pair-group method with arithmetic mean (UPGMA) clustering tree is a commonly used method for sample similarity clustering. UPGMA clustering trees at the phylum, genus, and species levels intuitively display the genetic relationship and grouping pattern of flora between groups, providing key evidence for the analysis of flora structural differences. Compared with the NC group, the relative abundances of intestinal flora in the other groups exhibited significant differences at the phylum, genus, and species levels.

**FIGURE 3:**
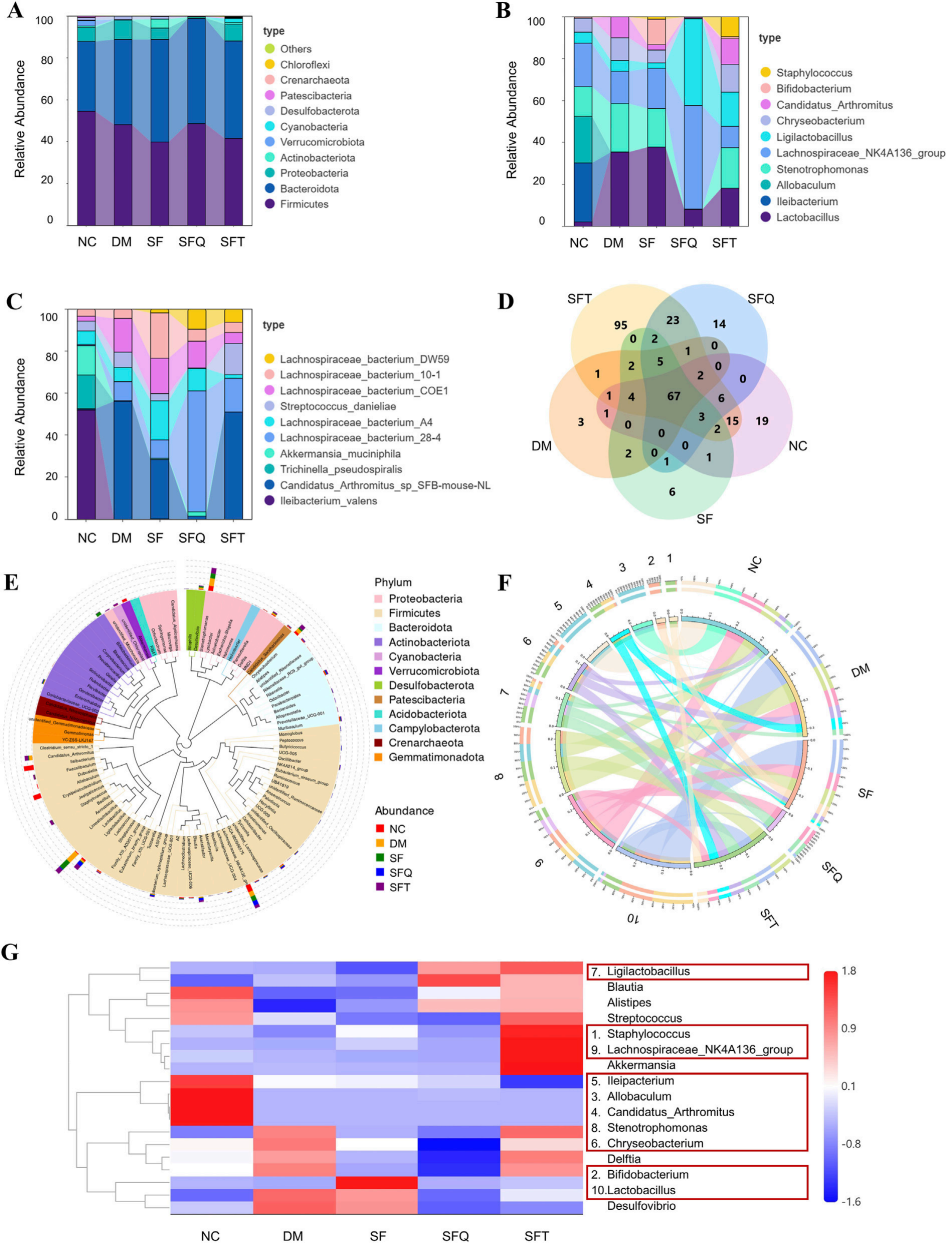
Different groups mice show significant alterations in the composition of the gut microbiota compared with NC group mice. **(A–C)** Differences in the gut microbiota among NC, DM, SF, SFQ and SFT group mice at the phylum, genus, and species levels. **(D)** Venn diagram shows the number of shared, unique, and depleted ASVs among five groups of mice at the genus level. **(E)** Phylogenetic tree of gut microbiota at the genus level. **(F)** Circos plot of the sample-gut microbiota species relationships at the genus level. **(G)** Clustering heatmap shows relative abundances of the gut microbial genera at the genus level.

At the phylum level, Firmicutes and Bacteroidetes were the absolutely dominant phyla in all groups. In addition, Verrucomicrobiota accounted for approximately 2.77% in the NC group and was almost undetectable in the other groups. Desulfobacterota (1.53%) and Proteobacteria (9.03%) were significantly elevated in the DM group. Actinobacteriota was significantly elevated in the SF (4.14%), SFQ (0.72%), and SFT (0.55%) groups (Figure 3A; Supplementary Figure S2D).

At the genus level, compared with the NC group, *Ileibacterium* and *Allobaculum* were almost undetectable in the other groups. *Stenotrophomonas* was almost undetectable in the SFQ group, while Lachnospiraceae_*NK4A1366_group* (4.67%) and *Ligilactobacillus* (3.93%) were significantly more abundant in the SFQ group than in the other groups (Figure 3B; Supplementary Figure S1F).

At the species level, the abundances of *Candidatus_Arthromitus_sp_SFB-mouse-NL* and *Streptococcus danieliae* were significantly elevated in the DM group, and the SFQ group significantly reversed this change. *Akkermansia muciniphila* was almost undetectable in the DM group but was effectively improved in the SF and SFQ groups. Lachnospiraceae*_bacterium_A4* and Lachnospiraceae*_bacterium_10-1* showed the highest expression abundances in the SF group, while Lachnospiraceae*_bacterium_28_4* and Lachnospiraceae*_bacterium_DW59* had the highest expression abundances in the SFQ group (Figure 3C; Supplementary Figure S2E).

Genus-level analysis revealed 67 shared amplicon sequence variants (ASVs) among the five groups (Figure 3D); 11 at the phylum level (Supplementary Figure S3A) and 25 at the species level (Supplementary Figure S2B). To further analyze the evolutionary relationships of species at the genus level, multiple sequence alignment was performed on the top 100 genera by abundance to obtain representative sequences and construct a genus-level species phylogenetic tree (Figure 3E). In addition, the top 10 species by abundance at the phylum, genus, and species levels were selected to draw sample-species relationship circular plots (Figure 3F; Supplementary Figure S3C,D) and species abundance clustering heatmaps (Figure 3G; Supplementary Figure S3E,F), respectively.

### 3.5 Statistical analysis of intestinal flora differences

**FIGURE 4:**
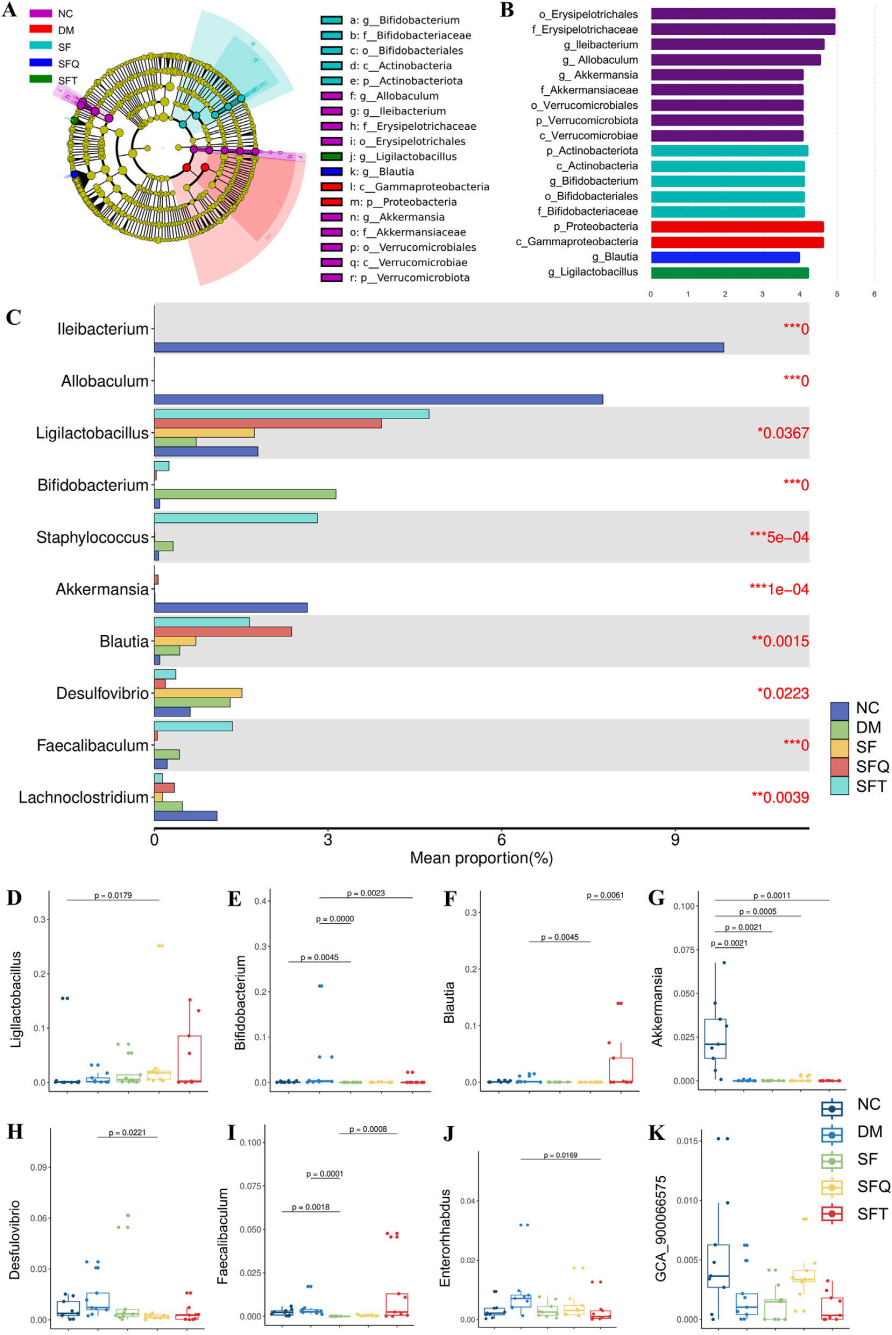
Statistical tests to evaluate differences in the gut microbiota community structure among different groups. **(A)** LEfSe evolutionary branch diagram. **(B)** Bar chart of LDA value distribution. **(C–K)** Top 10 microbial genera exhibiting significant differences among five groups. Kruskal-Wallis H test and Dunn’s test with Holm correction were performed among five groups; six mice per group; Error bars represent standard deviation.

The LEfSe cladogram (phylogenetic distribution, Figure 4A) and LDA value distribution histogram (LDA >4, p < 0.05, Figure 4B) showed that each group had distinct signature flora with significant differences. The NC group was characterized by core beneficial bacteria that produce butyrate and protect the intestinal barrier, including Erysipelotrichaceae, Verrucomicrobiota, *Akkermansia*, *Ileibacterium*, and *Allobaculum*; while the DM group was significantly enriched with Proteobacteria and Gammaproteobacteria. The SF group was marked by *Ligilactobacillus* and the SFT group by *Bifidobacterium*, while the signature bacterium of the SFQ group was *Blautia*. The LDA scores of multiple differential flora within the same group were similar, indicating that they jointly constitute the core signature flora of each group and make important contributions to the intergroup differences in community structure. We also performed intergroup differential abundance analysis at the genus level using the Kruskal-Wallis H test and Dunn’s test with Holm correction, identifying the top 10 species with significant intergroup differences (p < 0.05) (Figure 4C) and clarifying the specific expression differences between each group (Figures 4D–K).

### 3.6 QSYQ potentially modulated the impaired functional of intestinal flora

**FIGURE 5:**
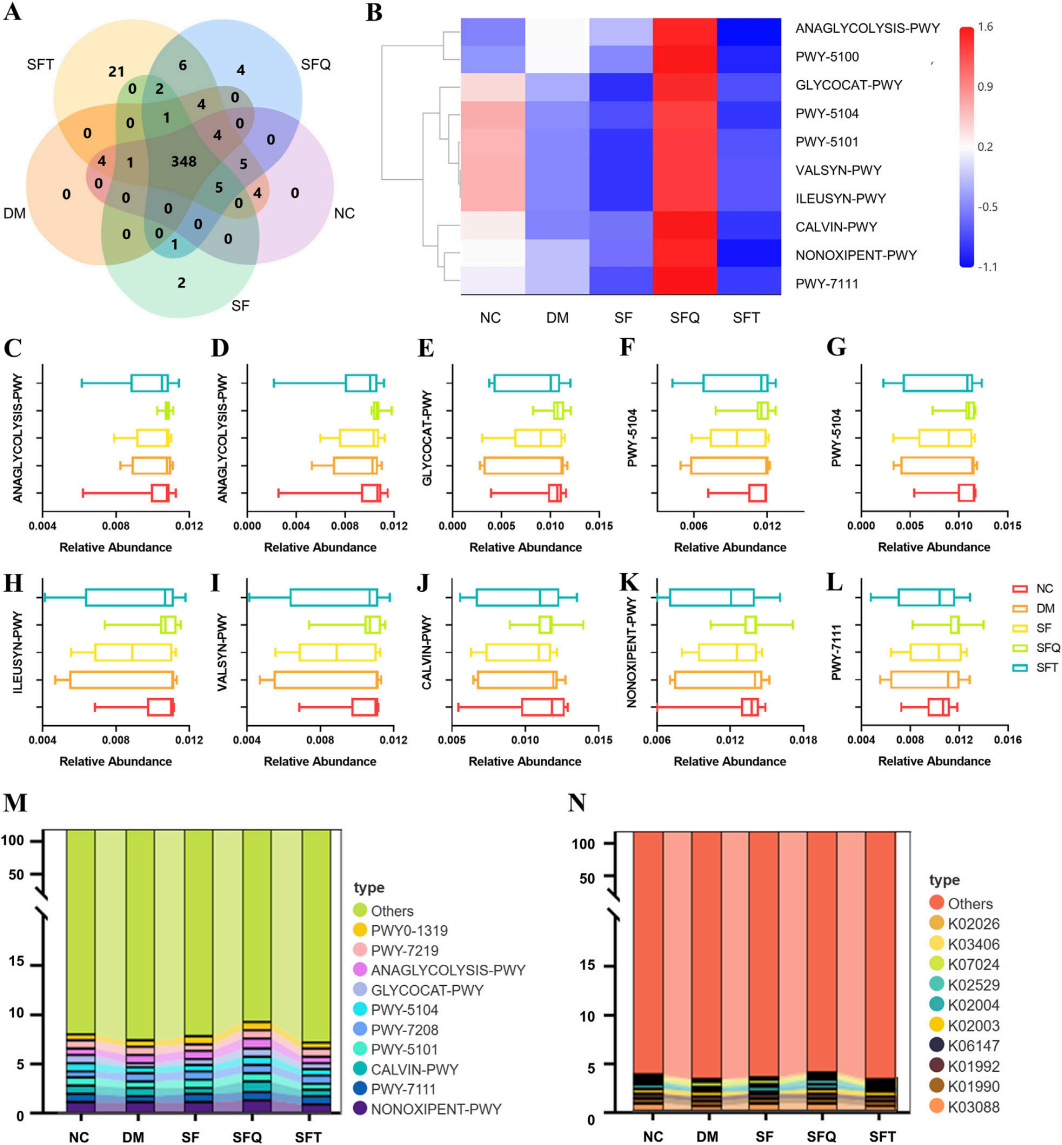
PICRUSt2 functional prediction of differential gut microbial genera. **(A)** Venn diagram of the number of shared, unique, and depleted functional pathways among five groups of mice. **(B–L)** Heatmap and box plot of Top 10 PICRUSt2 functional annotation of the gut microbiota based on the KEGG pathways. Kruskal-Wallis H test and Dunn’s test with Holm correction were performed among five groups; six mice per group; Error bars represent standard deviation. **(M, N)** Bar charts visualized the composition and distribution of pathway types and KO terms across all groups.

Based on the Kyoto Encyclopedia of Genes and Genomes (KEGG, https://www.kegg.jp/) and the MetaCyc metabolic pathway database (https://metacyc.org/), PICRUSt2 functional annotation tool was used to predict the metabolic functions of differential genera, and 348 shared pathways (PWY) were identified among the five groups (Figure 5A). We selected the top 10 metabolic pathways by genus-level abundance for cluster analysis (Figure 5B) and calculated the specific abundance differences between each group (Figures 5C–L). The results showed that pathways such as ANAGLYCOLYSIS-PWY, PWY-5100, GLYCOCAT-PWY, PWY-5104, PWY-5101, VALSYN-PWY, ILEUSYN-PWY, CALVIN-PWY, NONOXIPENT-PWY, and PWY-7111 are mainly involved in energy metabolism processes including the tricarboxylic acid cycle, pentose phosphate pathway, glycolysis pathway, and amino acid biosynthesis pathway. These processes may represent an adaptive response to disease-induced intestinal hypoxia, oxidative stress, and nutritional imbalance. QSYQ may be associated with inflammatory injury by remodeling the microenvironment and promoting host amino acid metabolism. We also compared and displayed the expression trends of the top 10 PWY and KO pathways in each group (Figures 5M,N). MetaNet species modular association network analysis suggested the potential interaction patterns of different species in biological systems and intuitively by constructing and accurately quantifying species association networks, thereby exploring the biological significance behind species abundance data (Supplementary Figure S4A-E). Analysis based on topological indicators such as modularity and average path length (Supplementary Table S1) could predict that the intestinal flora of the DM group had poorer stability, lower metabolic efficiency, and defected in anti-interference ability and functional synergy, which provided a possible basis for the repair of functional modules by QSYQ.

### 3.7 Quantitative analysis and pathway annotation of metabolomics in mouse intestinal contents

**FIGURE 6:**
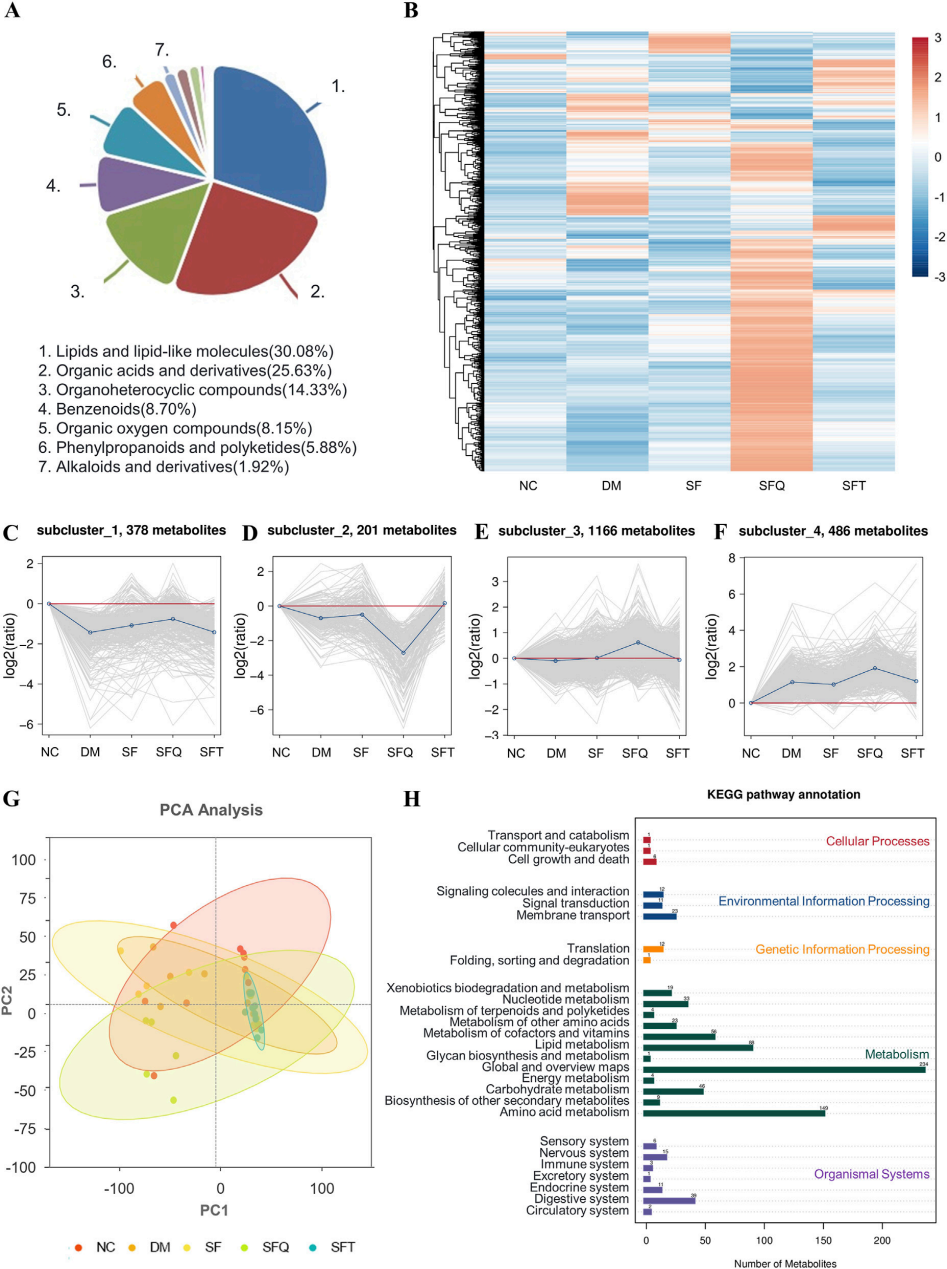
Quantitative analysis and pathway annotation of metabolomics from intestinal contents. **(A)** Pie chart of metabolite Class I classification. **(B–F)** Heatmap and K-means Cluster analysis of differential metabolites. **(G)** PCA analysis. **(H)** KEGG pathway enrichment and annotation of differential metabolites.

Untargeted metabolomic detection of intestinal contents was performed in mice of each group, and a total of 4320 metabolites were identified. Class I classification analysis showed that the top seven metabolite categories by proportion were Lipids and lipid-like molecules (30.08%), Organic acids and derivatives (25.63%), Organoheterocyclic compounds (14.33%), Benzenoids (8.70%), Organic oxygen compounds (8.15%), Phenylpropanoids and polyketides (5.88%), and Alkaloids and derivatives (1.92%) (Figure 6A).

K-means heatmap (Figure 6A) and principal component analysis (PCA) (Figure 6B) cluster analyses based on differential metabolites in mice of each group revealed that compared with the NC group, the expression levels of 1745 metabolites were decreased in the DM group and partially increased in the SF group, among which 1544 metabolites showed a more significant reversal in expression levels in the SFQ group (Figures 6C–F). The KEGG pathway database (https://www.genome.jp/kegg/pathway.html) classifies biological metabolic pathways into three hierarchical levels. Data from this study showed that amino acid metabolism (149) and lipid metabolism (88) were the two most highly enriched metabolic pathways (Figure 6H).

### 3.8 Correlation analysis of differential intestinal flora and differential metabolites

**FIGURE 7:**
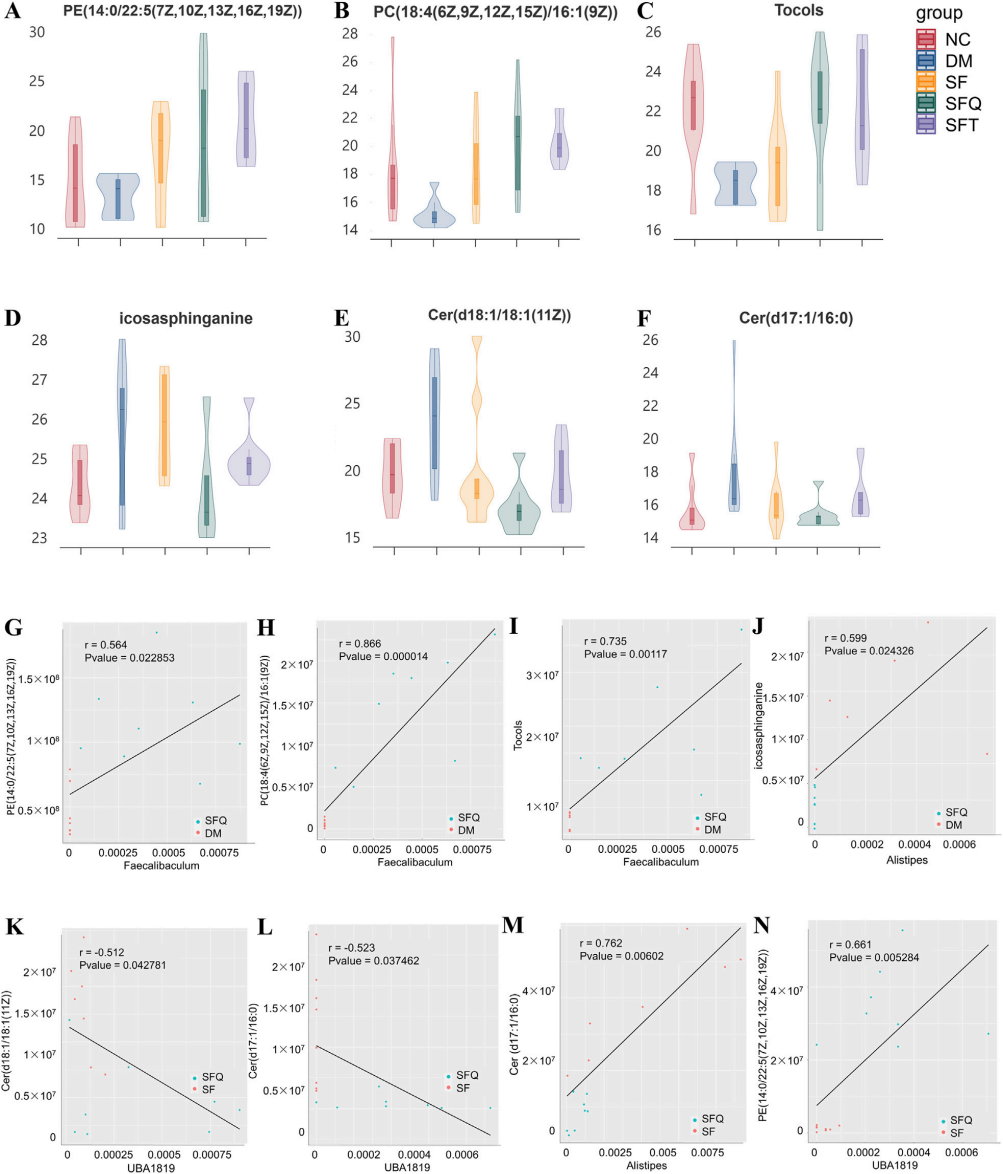
Correlation analysis between differential gut microbiota and differential metabolites in four groups of mice. **(A–F)** The differential expression of PE (14:0/22:5 (7Z,10Z,13Z,16Z,19Z)), PC(18:4 (6Z,9Z,12Z,15Z)/16:1 (9Z)), Cer(d17:1/16:0), Cer(d18:1/18:1 (11Z)), Tocols and icosasphinganine. **(G–N)** Scatter plot shows the top eight pairs (largest |r| values) of differential genera and differential metabolites based on the Pearson correlation coefficient (r) and *P*-value.

Pearson correlation analysis was performed between the most significantly differential genera at the genus level in 16S rDNA sequencing and the top 10 most significantly differential metabolites in metabolomics. We selected four comparison pairs (DM vs. NC, SF vs. DM, SFQ vs. DM, SFQ vs. SF) to draw heatmaps (Supplementary Figure S5A-D) for analyzing the correlation between microbial diversity and differential metabolites in the samples. Based on this, we screened six characteristic metabolites with differential trends in these comparison pairs (Figures 7A–F), which were found to belong to glycerophospholipids, tocols (vitamin E compounds), sphingolipids and their precursors, and are widely involved in cell membrane structure maintenance, oxidative stress, inflammatory response, and lipid signaling pathways. Combined with microbial data analysis results, the Pearson correlation coefficients (r) and p-values between differential genera and differential metabolites were calculated, and scatter plot correlation analysis was performed for metabolite-flora pairs with biological significance (Figures 7G–N). The results showed that *Faecalibaculum*, *UBA 1819*, and *Alistipes* exhibited changes in several comparison pairs. *Faecalibaculum* was positively correlated with PE (14:0/22:5 (7Z,10Z,13Z,16Z,19Z)), PC(18:4 (6Z,9Z,12Z,15Z)/16:1 (9Z)), and tocols in the SFQ group; *Alistipes* was positively correlated with icosasphinganine and Cer(d17:1/16:0) in the DM and SF groups, respectively; *UBA1819* was negatively correlated with Cer(d17:1/16:0) and Cer(d18:1/18:1 (11Z)) and positively correlated with PE (14:0/22:5 (7Z,10Z,13Z,16Z,19Z)) in the SFQ group. Each drug-administered group may be suggestive of enhancing the ability to resist oxidative stress injury and restore the integrity and fluidity of cell membranes by improving the abundance of Faecalibaculum in the intestinal flora; and promoting intestinal barrier repair and inflammatory homeostasis remodeling by reducing the abundances of *Alistipes* and *UBA1819* and decreasing the content of ceramide substances.

### 3.9 Potential mechanisms underlying the protective effects of QSYQ

**FIGURE 8:**
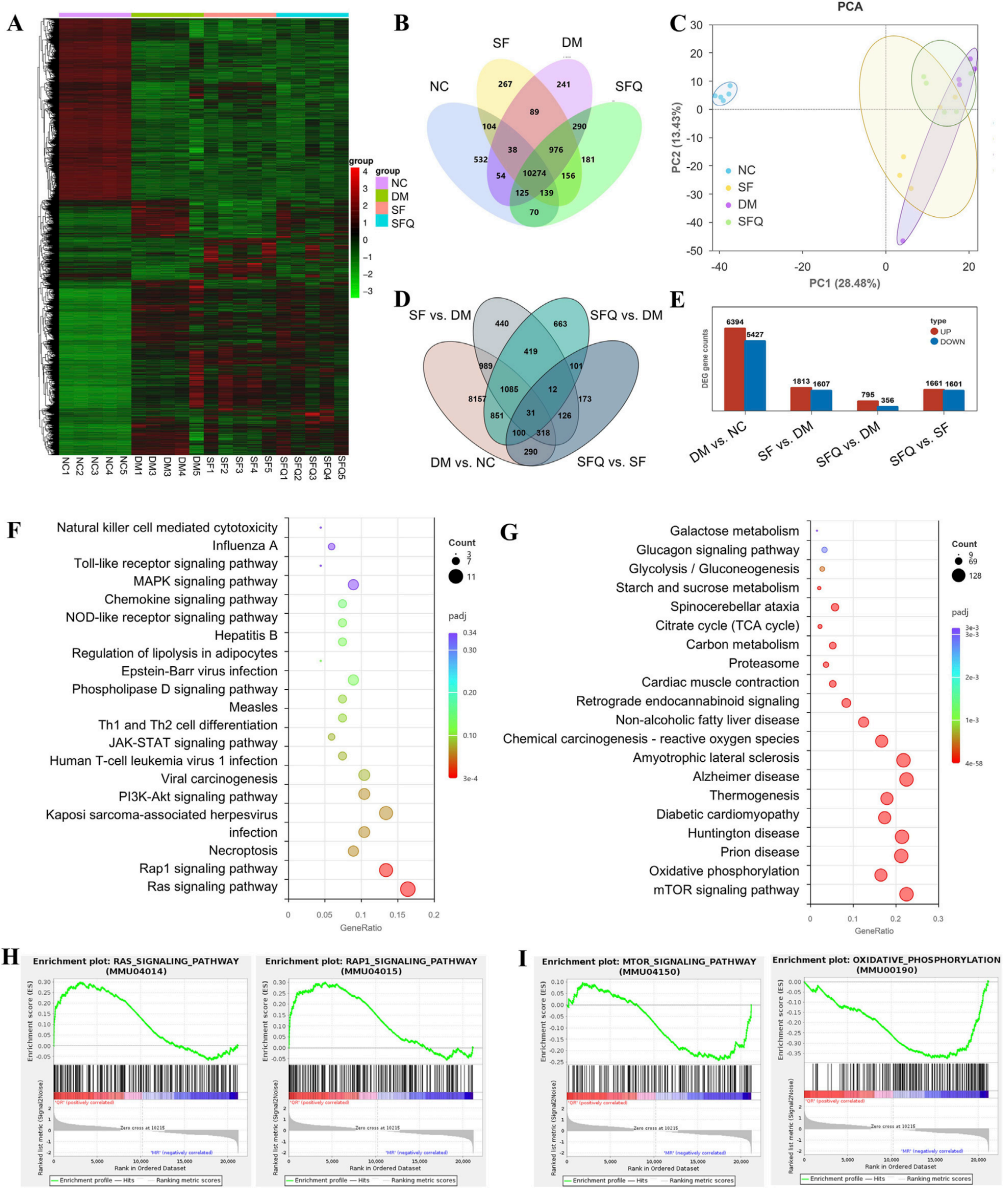
Quantitative analysis of muscle transcriptomics and functional enrichment analysis. **(A)** Transcriptomic heatmap of mouse skeletal muscle tissue. **(B)** Venn diagram of the number of shared, unique, and depleted genes among NC, DM, SF and SFQ groups of mice **(C)** PCA analysis. **(D,E)** Venn diagram of differential genes and DEG gene counts among four pairs of comparisons, including DM vs. NC, SF vs. DM, SFQ vs. DM and SFQ vs. SF. **(F,G)** Bubble plot presenting top enriched KEGG pathways for DEGs from SFQ and SF groups comparisons. **(H,I)** GSEA enrichment plots of top enriched pathways.

To reveal the potential mechanisms by which QSYQ exerts protective effects on skeletal muscle and liver functions, transcriptomic analysis was performed on skeletal muscle tissues of mice in the NC, DM, SF, and SFQ groups (Figure 8A). The results showed that 10,274 genes were expressed in all samples of the four groups (Figure 8B), with significant differences in the overall gene distribution (Figure 8C). A total of 31 differentially expressed genes (DEGs) were expressed in all four comparison pairs (DM vs. NC, SF vs. DM, SFQ vs. DM, SFQ vs. SF) (Figure 8D). The specific upregulation and downregulation of gene expression between each group are detailed in Figure 8E.

KEGG functional enrichment bubble figures (Figures 8F,G) shows the top enriched pathways were mainly clustered into Ras and Rap1 signaling pathways in SF group while mTOR and Oxidative Phosphorylation in SFQ group. Each pathway exhibited distinct GeneRatio and adjusted p-value (padj) distributions in GSEA analysis (Figures 8H,I).

**FIGURE 9:**
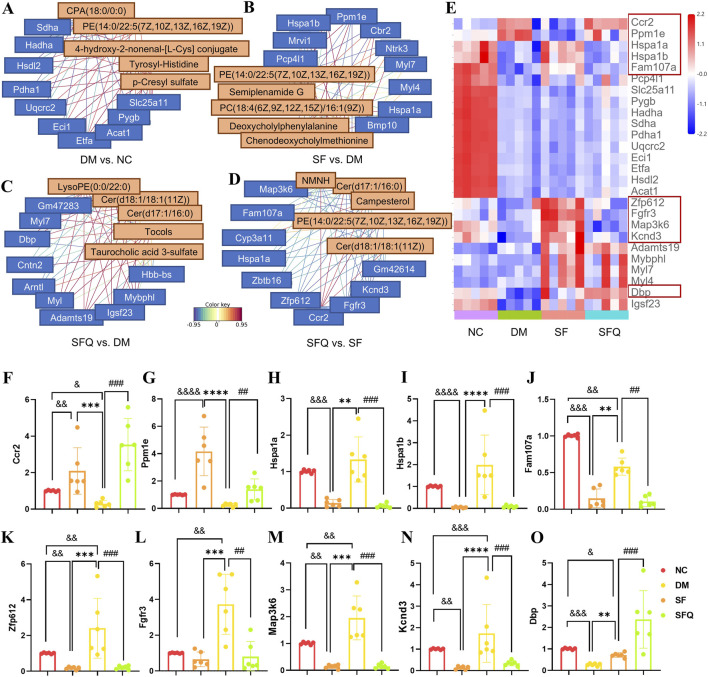
Correlation analysis and functional enrichment about differential genes and differential metabolites in four groups of mice. **(A–D)** Network diagram of the correlation analysis between differential gut microbiota and differential metabolites of above four pairs of comparisons. The value range of the correlation coefficient was (−0.95, 0.95). **(E)** Heatmap of ten hub candidate genes across NC, DM, SF and SFQ groups. The color gradient corresponds to standardized gene expression values, with red representing upregulation and blue representing downregulation. **(F–O)** qPCR validation of the relative mRNA expression of the ten hub genes (Ccr2, Ppm1e, Hspa1a, Hspa1b, Fam107a, Zfp612, Fgfr3, Map3k6, Kcnd3, Dbp). Six mice per group, all data are expressed as mean ± standard deviation. One-way analysis of variance (ANOVA) was used for statistical analysis. &*p* < 0.05, &&*p* < 0.01 compared with NC group, ^✱^ *p* < 0.05, ^✱✱^ *p* < 0.01 compared with DM group, #*p* < 0.05, ##*p* < 0.01 compared with SF group.

We selected the top 10 most significantly differential genes in each comparison pair and the top five most significantly differential metabolites in metabolomics for Pearson correlation analysis (Figures 9A–D). The union of these differential genes was used to draw a heatmap to observe the changes in their expression levels in each group (Figure 9E). Gene Ontology (GO) Analysis showed up to 10 significantly enriched terms in the biological process (BP), cellular component (CC), and molecular function (MF) categories, respectively (Supplementary Figure S6A). qPCR validation revealed that statin treatment (SF group) induced a characteristic pattern of dysregulated gene expression: the damage-related genes *Hspa1a, Hspa1b, Fam107a, Zfp612, Fgfr3, Map3k6,* and *Kcnd3* were significantly upregulated, while the immune and metabolic regulatory genes *Ccr2, Ppm1e,* and *Dbp* were significantly downregulated. Notably, QSYQ treatment (SFQ group) effectively reversed both patterns.

Among these, the most critical enrichments were mainly associated with mitochondrial structure and energy metabolism, lipid metabolism, and stress response, which may be closely related to the efficacy of QSYQ in improving glucose and lipid metabolism and protecting hepatic and skeletal muscle function.

## 4 Discussion

Plenty of evidence-based research has confirmed that statins therapy, as a first-line cardiovascular intervention, exhibits favorable safety profiles and clinical benefits (Ray et al., 2022; Arvanitis and Lowenstein, 2023). However, in recent years, the side effects of statins have garnered increasing clinical attention (Ruscica et al., 2023). Notably, several studies have demonstrated that long-term statin administration exacerbates insulin resistance, leading to a significant elevation in blood glucose and aggravated renal inflammation and fibrosis (HUANG et al., 2023). Fenofibrate may reduce the risk of adverse cardiovascular events in patients with T2DM through multiple mechanistic pathways and confers certain benefits for glycemic improvement (Ferreira et al., 2022; Cao et al., 2020). Therefore, in the present study, a combination regimen of simvastatin and fenofibrate was adopted for the treatment group of mice with comorbid T2DM and CHD. Nevertheless, our findings still indicated that simvastatin-fenofibrate combination therapy yielded limited benefits for insulin resistance and abnormal glucose metabolism, and potentially induced skeletal muscle adverse reactions and hepatic function impairment. This finding is consistent with the conclusions of recent studies on statin-associated muscle symptoms (SAMS) (Hou et al., 2024). Consequently, the present study recommends regular monitoring of blood glucose, hepatic and renal function in patients with comorbid T2DM and CHD to mitigate the risks associated with long-term statin use.

QSYQ, a compound traditional Chinese medicine, had been demonstrated that the major chemical components we used were consistent with the Chinese Pharmacopoeia in our laboratory (Chinese Pharmacopoeia Commission, 2025; Zhan et al., 2025). QSYQ can improve mitochondrial respiratory chain electron transport, inhibit oxidative stress-induced injury, and exert a prominent cardioprotective effect, thus being widely used in clinical cardiovascular practice (Zheng et al., 2019). Endorsed by the China Association of Chinese Medicine (CACM), the expert consensus on the clinical application of QSYQ has been formally promulgated under the document identifier GS/CACM 366-2024. Preclinical studies have also verified that QSYQ can ameliorate diabetic nephropathy (Li and Xu, 2022).

Our experimental results confirmed that QSYQ exerts significant efficacy in reducing elevated fasting blood glucose (FBG), improving abnormal glucose tolerance and insulin resistance, as well as protecting skeletal muscle tissue and the liver from statin-induced toxic injury. The SFQ group was enriched with the relative abundance of microbial genera commonly considered beneficial including *Bifidobacterium,* Lachnospiraceae*, Blautia* and *Akkermansia* (Gurung et al., 2020; Qi et al., 2022; Kim et al., 2022), all of which have been validated to be negatively correlated with T2DM. While the F/B ratio roughly reflects gut dysbiosis, detailed data showed SF treatment raised harmful pro-inflammatory bacteriota and QSYQ supplementation relieved these flora disturbances by enriching metabolic friendly commensals, suggesting QSYQ may modulate lipid-lowering drug induced gut microecological imbalance. Intestinal microbiota merely serves as a single regulatory layer. Its changing patterns vary markedly across phylum and genus levels, and divergent analytical pipelines may also yield inconsistent outcomes. Hence, microbial trends can only act as auxiliary references rather than definitive evidence.

Collectively, these genera potentially exerted protective metabolic effects by maintaining intestinal barrier integrity and producing short-chain fatty acids. These microbial changes were associated with improved performance on inhibiting oxidative stress and inflammatory responses (Wu et al., 2025). Nevertheless, the present study has several inherent limitations worthy of clarification. For instance, we only characterized the correlation between intestinal flora and circulating metabolites at the observational level, without causal validation via fecal microbiota transplantation (FMT) or metabolite supplementation experiments to get targeted functional verification (Cheng et al., 2026). Meanwhile, our study suggested that the regulation of skeletal muscle transcriptomic gene expression by QSYQ mainly focused on pathways related to cell structure, energy metabolism, and oxidative stress, which is consistent with the mechanism by which QSYQ exerts its effects in myocardium. Furthermore, QSYQ may also be beneficial to muscle fibrosis in mice with abnormal glucose and lipid metabolism by regulating several transcription factors (Lu et al., 2021). Final phenotypes related to skeletal muscle and systemic metabolism are jointly governed by intestinal metabolites, intestinal barrier integrity, circulating bioactive substances, and skeletal muscle transcriptional networks. Fluctuations in the abundance of a single genus cannot perfectly align with measurements from isolated tissues or serum samples, which inherently explains the partial mismatches observed between microbial profiles and terminal phenotypic readouts.

Another key finding of the present study is the recognition that heterogeneity in the composition of intestinal flora and metabolites not only exists during the pathological progression of T2DM and CHD, but can also be induced by certain clinical therapeutic interventions. Most current research has focused on the beneficial pharmacological effects of first-line clinical agents such as statins and fibrates (Reichel and Knauf, 2021; Tardo et al., 2025), whereas their adverse side effects have not been fully investigated and elucidated. In this study, we explored the potential links underlying these drug-induced side effects from the perspective of intestinal flora and their metabolites, suggestting the potential beneficial regulatory effects of glycerophospholipids and tocols as well as the detrimental effects of ceramides in the comorbid T2DM and CHD model (Li et al., 2025a). Consistent with recent reports, statins alleviate vascular oxidative stress and inflammation to stabilize atherosclerotic plaques by triggering Nrf2 activation downstream of PI3K/Akt and ERK signaling (Marzoog and Kopylov, 2026). This regulatory axis overlaps with the previously validated protective mechanisms of QSYQ in our lab, offering a promising direction for our subsequent mechanistic research. We tried to explore the potential network among therapeutic drugs, the intestinal microbiota and clinical metabolic factors. We believe further work may help identify novel therapeutic targets for diabetes management, deepening the mechanistic understanding of how the intestinal microbiota modulates the comorbidity of T2DM and CHD, and facilitating the diversification of clinical intervention strategies for this complex comorbid condition.

## 5 Conclusion

The present study showed that drugs could effectively alleviated hyperlipidemia in ApoE^−/−^ mice with comorbid T2DM and CHD, with QSYQ exhibiting superior efficacy in regulating glucose metabolism, improving insulin resistance and protecting hepatic and skeletal muscle function. These protective effects of QSYQ may be associated with intestinal microbiota structure, metabolites, and modulated expression of inflammation, apoptosis, antioxidant and energy metabolism-related genes, offering a novel complementary therapeutic option for clinical management of T2DM combined with CHD.

## Data Availability

The datasets presented in this study can be found in online repositories. The names of the repository/repositories and accession number(s) can be found in the article/Supplementary Material.
