# Supplementary material for: Effects of QiShenYiQi dropping pills on gut dysbiosis and statin-associated muscle symptoms in ApoE−/− mice with type 2 diabetes mellitus and coronary heart disease
Source: Front Cell Dev Biol. 2026 Jul 27;14:1837554. doi: 10.3389/fcell.2026.1837554 (PMC13454115; doi:10.3389/fcell.2026.1837554)
Supplement: Supplementary file 1 [file DataSheet1.docx]

**Supplementary Material**

1. **Supplementary Methods**
2. **Supplementary Figures**
3. **Supplementary Tables**

**Supplementary Table 1**

**Supplementary Table 2**

1. **Supplementary Methods**
   1.1 **Animals and groups**

After one week of adaptive feeding, all 30 experimental mice were randomly divided into two batches. Six mice were assigned to the normal control (NC) group and maintained on a standard chow diet throughout the entire experimental period. The remaining 24 mice were subjected to combined modeling of T2DM combined with coronary heart disease and statin-associated muscle injury. Briefly, mice were fed a high-fat diet (HFD) for 30 consecutive days. From day 25 to day 27 of modeling, streptozotocin (STZ, Beyotime, Shanghai, China) dissolved in sterile normal saline was intraperitoneally injected at a dose of 100 mg/kg per day. Fasting blood glucose (FBG) was dynamically monitored to verify successful modeling of glucose metabolism disorder.

On day 30 after HFD feeding, the 24 successfully modeled mice were randomly divided into four groups (n = 6 per group): disease model group (DM), simvastatin combined with fenofibrate group (SF), simvastatin + fenofibrate + QSYQ group (SFQ), and simvastatin + fenofibrate + Trimetazidine group (SFT). All drug interventions were administered via intragastric gavage once daily for 60 consecutive days. The dosage of each drug was determined according to previous validated preclinical studies and equivalent body surface area conversion in mice.

Mice in the NC and DM groups received an equal volume of sterile normal saline gavage. The SF group was treated with simvastatin and fenofibrate to induce statin-associated muscle symptoms (SAMS). The SFQ group was given QSYQ standardized preparation combined with dual lipid-lowering drugs, while the SFT group received the positive control drug combined treatment. Detailed grouping information, intervention regimens and drug dosage standards are illustrated in Figure 1A.

We adopted a five-layer hierarchical grouping design (normal control NC, untreated comorbid model DM, simvastatin-fenofibrate combination SF, SF plus QSYQ SFQ, and SF plus trimetazidine positive control SFT). This grouping strategy clearly distinguishes two sources of tissue injury: comparison between DM and NC groups reveals intrinsic metabolic and organic damage originating from T2DM-CHD comorbidity, while comparison between SF and DM groups separately identifies extra muscular and hepatic toxicity triggered by combined statin-fibrate treatment. The SFQ and SFT groups further evaluate the synergistic protective efficacy of two candidate agents against both disease-derived lesions and drug-induced toxicities, which effectively resolves the confounding challenge of multiple overlapping variables in this compound disease model.

1.2 **16S rRNA Sequencing and Gut Microbiota**

All 16S rRNA amplicon sequencing services were completed by Novogene Co., Ltd. (Beijing, China). Total microbial genomic DNA was extracted from mouse fecal specimens. The hypervariable V4 region of the bacterial 16S rRNA gene was amplified with universal barcoded primers 515F (GTGCCAGCMGCCGCGGTAA) and 806R (GGACTACHVGGGTWTCTAAT), and purified PCR products were constructed into sequencing libraries and subjected to paired-end sequencing on the Illumina NovaSeq 6000 platform with a target sequencing depth of no less than 50,000 clean reads per sample. Raw reads were quality-filtered to remove low-quality sequences with an average Phred quality score <20, followed by sequence assembly, quality filtering and chimera elimination; clean data were imported into QIIME2 pipeline to generate amplicon sequence variants (ASVs) with taxonomic annotation. We characterized gut microbial ecology via alpha and beta diversity analysis. Kruskal–Wallis H test and other statistical tools were adopted to identify intergroup microbial differences, and PICRUSt2 was used to predict potential functional profiles of differential flora.

1.3 **Untargeted Metabolomics Sequencing**

Untargeted metabolomic LC-MS detection was completed by Novogene Co., Ltd. (Beijing, China). Intestinal content samples from each group were ground with liquid nitrogen, extracted using pre-cooled 80% methanol, and subjected to low-temperature centrifugation to obtain metabolite supernatants. Equal volumes of all individual samples were pooled to prepare quality control (QC) samples for monitoring instrument stability throughout detection.

Metabolite separation was carried out on an ACQUITY UPLC HSS T3 column (1.8 μm, 2.1 mm × 100 mm) with a mobile phase consisting of A (0.1% formic acid in water) and B (0.1% formic acid in acetonitrile) at a flow rate of 0.3 mL/min; the column oven temperature was maintained at 40 °C. Mass spectrometry acquisition was performed under both positive electrospray ionization (ESI⁺) and negative electrospray ionization (ESI⁻) modes on an Orbitrap mass spectrometer. The spray voltage was set to 3.8 kV for ESI⁺ and 3.2 kV for ESI⁻, with sheath gas and auxiliary gas flow rates of 35 and 12 arbitrary units, respectively.

After raw data format conversion, peak picking, retention time alignment, mass correction and noise filtering, all metabolite peak intensities were normalized by total ion current (TIC) for subsequent comparative analysis. Metabolite annotation was matched against the in-house Novogene metabolite database. Principal component analysis (PCA), k-means clustering and correlation analysis were applied to evaluate sample grouping and metabolite association patterns. Metabolic pathway enrichment analysis was further conducted to interpret the biological functions of differential metabolites.

1.4 **RNA-seq**

RNA-seq detection and library construction were completed by Novogene Co., Ltd. (Beijing, China). Based on the complete data covering biochemical indices, histopathological staining and in vivo muscle function assays, trimetazidine (SFT group) only slightly alleviates hepatic injury, with nearly no protective effects against statin-triggered skeletal muscle damage and no hypoglycemic efficacy. The core objective of skeletal muscle transcriptome sequencing was to screen unique molecular signatures underlying the muscle-protective effects of QSYQ. Accordingly, only the NC, DM, SF and SFQ groups were selected for RNA-seq library construction and sequencing, which reduces background noise from irrelevant transcripts and cuts unnecessary sequencing costs. Skeletal muscle tissues from each group (n = 5 biological replicates per group) were homogenized, and total RNA was extracted using TRIzol reagent following the manufacturer’s protocol. RNA integrity and purity were verified via agarose gel electrophoresis and Nanodrop quantification; only samples with RNA integrity number (RIN) > 6.8 proceeded to subsequent library preparation. Messenger RNA was enriched by magnetic oligo(dT) beads, fragmented randomly, and reverse-transcribed to generate cDNA libraries, which were sequenced as 150 bp paired-end reads on the Illumina HiSeq platform, with a target sequencing depth of no less than 6 Gb clean data per sample. Raw sequencing reads were quality-filtered to remove low-quality reads and adapter-contaminated sequences to yield clean reads, which were mapped to the mouse reference genome using HISAT2.

Differential expression analysis was performed with DESeq2. Raw P-values were corrected via the Benjamini–Hochberg algorithm to control false discovery rate. Genes satisfying adjusted P value < 0.05 and |log₂(fold change)| ≥ 1 were screened as differentially expressed genes (DEGs).

All DEGs were submitted to KEGG pathway enrichment analysis; pathways with adjusted P < 0.05 were regarded as significantly enriched functional pathways.

Gene set enrichment analysis (GSEA) was carried out on all expressed genes without pre-filtering DEGs, using KEGG gene sets as reference gene collections. The permutation number was set to 1000 times, and gene sets with normalized enrichment score (NES) |NES| ≥ 1.2 and nominal P < 0.05 were identified as significantly enriched pathways.

1.5 **Multi-Omics Correlation Analysis**

All sequencing datasets were preprocessed to acquire relative abundances of differential gut genera, quantitative levels of differential metabolites and normalized expression values of DEGs. Pearson correlation analysis was conducted pairwise among three omics layers to generate correlation coefficients (*r*) and corresponding raw *P* values. Multiple testing correction was implemented via the Benjamini–Hochberg FDR procedure to reduce false positive correlations. Correlation pairs satisfying FDR-adjusted *P* < 0.05 and |*r*| > 0.5 were retained for downstream visualization. Interaction network plots were constructed with the mixOmics package implemented in R.

2. **Supplementary Figures**

**Supplementary Figure S1. Mice in the AMI group exhibit alterations in the gut microbiota.**

(A-B) Venn diagrams of different groups at the phylum and species levels; (C-D) Circos plots show the relationships between samples and gut microbiota at the phylum and species levels; (E-F) Clustering heatmaps show Z-values of gut microbiota abundance at the phylum and species levels. As shown in Figures S1 C-F, the numerical labels in E and F are consistent with those in C and D, respectively.

**Supplementary Figure S2. The α-diversity and β-diversity indices of gut microbiota.**

(A) The species accumulation boxplots; (B) The rank abundance curves; (C) The rarefaction curve of chao1 (α-diversity indice); (D) Boxplots of the β-diversity index distribution; (E) The β-diversity Principal Coordinate Analysis (PCoA) based on the Bray-Curtis distance; (F) UPGMA clustering tree at the genus level. Kruskal-Wallis H test and Dunn's test with Holm correction were performed among five groups. *p < 0.05 vs. NC; 6 mice per group; Error bars represent standard deviation.

**Supplementary Figure S3. Gut microbiota α-diversity and β-diversity analysis.**

(A) The α-diversity indices and heatmaps of the Bray-Curtis distance matrices; (B) PCA; (C) NMDS; (D-E) UPGMA clustering trees at the phylum and species levels; (F-H) iCAMP, βNTI and NST. Kruskal-Wallis H test and Dunn's test with Holm correction were performed among five groups. *p < 0.05 vs. NC; 6 mice per group; Error bars represent standard deviation.

**Supplementary Figure S4. MetaNet species modular association network diagram of gut microbiota**

(A) NC group; (B) DM group; (C) SF group; (D) SFQ group; (E) SFT group. The overall network topology coefficients are shown in Supplemental Table 1. In this association network, each node represents a genus, and the size of the node indicates the relative abundance of the genus. The edge represents the functional-based positive or negative association between genera, with red edges indicating positive correlation and blue edges indicating negative correlation.

**Supplementary Figure S5. Heatmap for correlation analysis between differential gut microbiota and differential metabolites at genus level.**

(A) DM vs. NC group; (B) SF vs. DM group; (C) SFQ vs. DM group; (D) SFQ vs. SF group.

**Supplementary Figure S6.** **Differentially expressed genes from the correlation analysis between transcriptomics and metabolomics.**

(A) GO enrichment analysis and annotation. The cut-off of P-value is set to 0.05, terms of same category are ordered by P-values.
